# Antioxidant, Nutraceutical Properties, and Fluorescence Spectral Profiles of Bee Pollen Samples from Different Botanical Origins

**DOI:** 10.3390/antiox9101001

**Published:** 2020-10-15

**Authors:** Daniele Barbieri, Morena Gabriele, Martina Summa, Raffaele Colosimo, Donatella Leonardi, Valentina Domenici, Laura Pucci

**Affiliations:** 1 National Research Council (CNR), Institute of Agricultural Biology and Biotechnology (IBBA), Via Moruzzi 1, 56124 Pisa, Italy; danielebarbieri.bd@gmail.com (D.B.); morena.gabriele@ibba.cnr.it (M.G.); pucci@ibba.cnr.it (L.P.); 2 Chemistry and Industrial Chemistry Department, University of Pisa, Via Moruzzi 13, 56124 Pisa, Italy; m.summa1@studenti.unipi.it; 3 Quadram Institute Bioscience, Norwich Research Park, Norwich, Norfolk NR4 7UQ, UK; raffaele.colosimo@quadram.ac.uk; 4 Department of Biology, University of Rome “Tor Vergata”, Via della Ricerca Scientifica 1, 00133 Roma, Italy; leonardi@uniroma2.it

**Keywords:** bee pollen, phenolic composition, polyphenols, antioxidant activity, front-face fluorescence spectroscopy, UV-vis spectroscopy

## Abstract

Bee pollen is made by honey bees (*Apis Mellifera*) from the pollen of plants and flowers and represents an apiary product enriched in essential amino acids, polyphenols, omega-3, and omega-6 fatty acids. This study investigated the botanical origin, micronutrient profile, and antioxidant activity of bee pollen samples (*n* = 10) harvested in Lucca and Massa Carrara (Tuscany, Italy) between 2016 and 2017. The palynological analysis showed that bee pollen samples were composed of nine botanical families. Front-face fluorescence spectroscopy was performed on bee pollen samples in bulk, without any treatment, and in ethanol extracts to determine the characteristic fluorescent profile and, to identify the main chemical compounds with biological activity. The main chemical compounds detected were polyphenols (mainly flavonoids and phenolic acids), hydro-soluble vitamins (B_2_, B_3_, B_6_, and B_9_), amino acids, and pigments. Furthermore, the antioxidant activity was investigated, and one of the two *Viburnum* pollens resulted in the highest polyphenols and flavonoids content (20.15 ± 0.15 mg GAE/g fw and 23.46 ± 0.08 mg CE/g fw, respectively). However, *Prunus* and *Eucalyptus* families showed the highest in vitro (190.27 ± 8.30 µmol Fe^2+^/g) and ex vivo (54.61 ± 8.51 CAA unit) antioxidant capacity, respectively. These results suggested that Tuscan bee pollen, depending on the botanical family, is rich in essential nutrients and potential nutraceutical product.

## 1. Introduction

Honey bees (*Apis Mellifera*) harvest the pollen from plant flowers and enrich it with salivary enzymes and nectar [1] to obtain small granular-looking grains (bee pollen) that are transported into the apiary. Bee pollen is abundant and essential nourishment that could satisfy the protein needs of the entire colony [2,3]. A single pollen bead has a color that is due to its specific botanical origin. Therefore, it is possible to find bee pollen from one specific flower or belonging to several flower species. The first case is called monofloral, while the second case is called polyfloral. Moreover, it is possible to blend different monofloral samples in order to create mixtures of bee pollen with mixed organoleptic properties and attributes [4].

The chemical composition of bee pollen results in about two hundred compounds [5,6] of which several metabolites have the purpose of ensuring the preservation of the bee pollen (e.g., fat-soluble vitamins and polyphenols) [7]. The percentage of macronutrients (e.g., carbohydrates, proteins, and lipids), as well as the profile of the minor compounds (e.g., phenolic compounds), could vary in terms of quality and quantity, depending on botanical origin, but also climatic conditions, soil types, beekeeper’s activity, and preservation methods [8].

Bee pollen could be used as a dietary supplement and its commercial interest is growing due to its high nutritional value and medical properties such as hypolipidemic [9], anti-inflammatory [10], and antiallergic activity [11]. These effects on human health [12] have been correlated with the polyphenols content and chemical composition of bee pollen, regardless of the aforementioned wide species-specific variation of nutrients and beneficial compounds that it could contain [2]. Moreover, the antioxidant properties of bee pollen and its content in terms of bioactive compounds is essential in determining the nutraceutical properties of honey and propolis, too [13,14]. The main phenolic compounds of bee pollen are phenolic acids, mainly hydroxybenzoic, and flavanols, that can be present in their free forms or as glycoside’s derivatives [6,15].

Most of these compounds, such as aromatic amino acids, many polyphenols, water/lipid-soluble vitamins, and pigments, show intrinsic fluorescent properties and can be studied through fluorescence techniques [16]. Over the past twenty years, the interest for the application of non-destructive fluorescence spectroscopic techniques in the study of food matrices, especially using the front-face fluorescence (FFF) method, has constantly been growing [17]. This is mainly due to the advantages that this technique offers in terms of minimal preparation of the sample that allows analyzing samples in their “bulk state” and, hence, reduces the time of analysis, the possibility of matrix alteration and/or contamination. Moreover, the fluorescence spectroscopy has higher sensitivity when compared to other spectroscopic techniques [17,18] in terms of changes in chemical surrounding (i.e., pH, temperature, solvent, and chemical composition of the food matrix), conformational properties (in particular for macromolecules, such as proteins), and fluorophores minimum detectable concentration (up to the order of ppb). To date, the FFF method has been successfully used for the study of several food matrices [18], such as vegetable oil [19], milk and dairy products [20,21], wine [22,23], cereals [24,25], and honey [26,27,28,29,30]. However, only one study on bee pollen has been reported based on FFF spectroscopy [4].

Hence, the purpose of this work was to underpin the link between chemical composition, botanical origin, and biological properties of ten samples of Tuscan bee pollen. The fluorescence spectral profile of each sample (bulk state and ethanol extracts) was investigated by the FFF technique. A semi-quantitative spectral analysis of the experimental FFF profiles was applied to identify the specific fluorophores such as polyphenol acids, vitamins, and pigments. The overall fluorescence spectral features were correlated to the botanical origin of the bee pollen samples (i.e., bee pollen FFF fingerprint). Moreover, the total phenolics and flavonoids content, as well as the in vitro (FRAP) and ex vivo cellular antioxidant activity in red blood cells (CAA-RBC), of all bee pollen samples were investigated.

## 2. Materials and Methods

### 2.1. Chemicals and Reagents

All standards and reagents were of analytical grade. Folin-Ciocalteau reagent, sodium carbonate, gallic acid, sodium nitrite, aluminum chloride, sodium hydroxide, catechin, chloridric acid, phosphate buffer saline (PBS), 2,4,6-Tri(2-pyridyl)-s-triazine (TPTZ), ferric chloride hexahydrate, ferrous sulfate heptahydrate, quercetin, 2,2-azobis (2-amidinopropane) dihydrochloride (AAPH), and dichlorofluorescein diacetate (DCFH-DA) were purchased from Fluka-Sigma-Aldrich (Spruce St, Saint Louis, MO, USA). Absolute ethanol was purchased from VWR (Radnor, PA, USA). Methanol (>99%, Fluka-Sigma-Aldrich) was used to prepare the standard solutions. Standard solutions were prepared from solids: 4-hydroxybenzoic acid (99%, Fluka-Sigma-Aldrich), gallic acid (98%, Fluka-Sigma-Aldrich), vanillic acid (97%, Fluka-Sigma-Aldrich), β-carotene (99%, Fluka-Sigma-Aldrich), riboflavin (vitamin B_2_) (95%, Merck KGaA, Darmstadt, Germany), tryptophan (98%, Fluka-Sigma-Aldrich), syringic acid (98%, Fluka-Sigma-Aldrich), 3,4-dihydroxybenzoic acid (97%, Fluka-Sigma-Aldrich), caffeic acid (97%, Fluka-Sigma-Aldrich), *p*-coumaric acid (>98%, Fluka-Sigma-Aldrich), ferulic acid (99%, Fluka-Sigma-Aldrich), sinapic acid (>99%, Fluka-Sigma-Aldrich), quercetin (>98%, Fluka-Sigma-Aldrich), nicotinic acid (vitamin B_3_) (98%, Fluka-Sigma-Aldrich), and pyridoxine (vitamin B_6_) (95%, Fluka-Sigma-Aldrich).

### 2.2. Bee Pollen Samples and Palynological Analysis

Five bee pollen samples *(Apis Mellifera*), named as P01, P02, P03, P04, and P05, were harvested in 2016 and 2017 from two different geographical areas of Tuscany (Italy), in particular Garfagnana (LU) and Caniparola-Fosdinovo (MS), by two different Tuscan farms, specifically “Apicoltura Biologica Aldo Metalori” (Massa Macinaia, LU, Italy) and “Azienda Apistica Guidarelli Andrea” (Fivizzano, Massa Carrara, MS, Italy). Among these five samples, composed by a mix of colored grains, bee pollens were divided based on the grain color and only those presenting a distinct homogeneous color were analyzed to confirm their monofloral character. The list of bee pollen samples, further divided for their botanical origin, is reported in Table 1. All samples underwent a light heat treatment (T_max_ = 38 °C) to reduce the free water content before being stored at −20 °C in the dark until further analysis.

The botanical origin of the bee pollen samples was confirmed by the melissopalynology analysis by means of an optical microscope. Each single pollen load was washed with distilled water and fixed with glycerin jelly. Bee pollen grains identification was performed by optical microscope (Leica DME, Buccinasco (MI), Italy) with total magnification (400X and 1000X). A reference collection of Tor Vergata University and different pollen morphology guides were used for the recognition of pollen [31].

### 2.3. Bee Pollen Extraction and Phytochemical Composition

Bee pollen grains were finely powdered with mortar and pestle. The extracts (50 mg/mL) were obtained after 1 h of incubation at room temperature in 95% ethanol while being gently stirred. Then, samples were centrifuged for 10 min at 3500 rpm at 4 °C, and the supernatants were collected and kept in the dark at 4 °C until use.

Total phenolics, estimated as Folin-Ciocalteau (FC) reducing capacity, were determined as reported by Gabriele et al. [4]. Briefly, 100 μL of ethanolic bee pollen extract was mixed with 500 μL of 0.2 N Folin-Ciocalteau reagent and incubated in the dark for 5 min. Then, 400 μL of 0.7 M sodium carbonate (Na_2_CO_3_) was added. The absorbance was recorded at 760 nm, after 2 h of incubation at room temperature in the dark. Gallic acid was used as a standard, and total phenolics were expressed as mg of gallic acid equivalents per g on a fresh weight basis (mg GAE/g fw).

Total flavonoids were determined using the aluminum chloride colorimetric method, as previously described by Gabriele et al. [4]. Briefly, 200 μL of ethanolic bee pollen extract was mixed with 800 μL of H_2_O and 60 μL of 5% NaNO_2_, followed by 5 min of incubation at room temperature. Finally, 60 μL of 10% AlCl_3_ were added, incubated for 6 min, and the reactions were neutralized with 400 μL of 1 M NaOH. Absorbance was measured at 430 nm after 30 min of incubation. Catechin was used as a standard, and flavonoids were expressed as mg of catechin equivalents per g on a fresh weight basis (mg CE/g fw).

### 2.4. UV-Visible Absorption Spectroscopy

UV-visible absorption spectra were acquired on each bee pollen ethanol extracts by means of a double beam UV-vis spectrophotometer Jasco V-550 (JASCO, Korea University, Seoul, Korea). About 1 mL of sample extract was put in a quartz cuvette for UV-vis analysis (with the optical path of 0.5 cm). Each spectrum was recorded between 220 and 750 nm with a scanning speed of 400 nm/s, a width of slits and an increment of 0.5 nm.

### 2.5. Front-Face Fluorescence Spectroscopy

Fluorescence investigations were performed by using an ISA Fluoromax II spectrofluorimeter (Horiba, Kyoto, Japan) equipped with a Xenon arc lamp and a cell holder device for front-face (reflectance) measurements. To this purpose, the cell position inside the cell-holder was set with an incident angle optimized at 31° (namely the angle between the incident excitation beam and the sample surface normal), to eliminate or reduce self-absorption effects, light reflection superposition and light scattering [32].

Each pollen sample was studied in its bulk state, without any chemical treatment, and as ethanol extracts, by front-face fluorescence spectroscopic techniques to study the specific spectral emission profiles and to recognize the main classes of fluorescent compounds in pollen grains. The identification of fluorophores was performed either by comparing the characteristic emission peaks with those of minor compounds whose fluorescence spectra are reported in the literature or by performing a semi-quantitative spectral analysis, based on the simulation of the experimental spectra, as described by Parri et al. [29,30].

For the bulk analysis, pollen loads were reduced in the form of powder using mortar and pestle. A little amount of powder (~10 mg) was put between two quartz windows of 1 mm optical path. These quartz windows were held against support in the spectrofluorometer cell-holder by a laminar spring. Emission spectra (with λ_ex_ ranging between 280 and 550 nm), excitation spectra (with λ_em_ ranging from 360 and 650 nm, depending on the sample, as described in Section 3) and synchronous spectra (with ∆λ ranging from 20 to 120 nm) were recorded for each sample. In all cases, the excitation and emission slits were fixed to 2.5 nm, the constant integration time at 0.5 s, and the wavelength increment was 1 nm. Before the spectral analysis, emission intensity was corrected, taking into account the light scattering contribution, estimated by using a light diffuser (dried Na_2_SO_4_) as previously reported [29,30,32].

In the case of ethanol extracts of the bee pollen samples, prepared as reported in Section 2.3, a quartz cell with 10 mm optical path was used to record emission (λ_ex_ = 280 nm, λ_ex_ = 320 nm, λ_ex_ = 350 nm, λ_ex_ = 410 nm, λ_ex_ = 430 nm), excitation (λ_em_ = 410 nm, λ_em_ = 430 nm, λ_em_ = 480 nm, λ_em_ = 530 nm), and synchronous spectra (∆λ = 70 nm, ∆λ = 120 nm) using the same instrumental parameter above-mentioned.

In all cases, the intensity of the FFF spectra was determined as the ratio between the emission signal (counts per seconds, cps) and the intensity of light from the excitation monochromator (mA), measured by means of a photomultiplier and a photodiode, respectively. In most of the cases, as reported in the text, spectra were normalized and arbitrary units (a.u.) used.

### 2.6. Determination of Bee Pollen In Vitro Antioxidant Activity

The antioxidant activity of ethanolic bee pollen samples was determined by FRAP assay as previously reported by Colosimo et al. [33]. Briefly, a freshly prepared FRAP solution (2500 μL) containing acetate buffer 300 mM (pH 3.6), TPTZ 10 mM in HCl 40 mM, and FeCl_3_·6H_2_O 20 mM at a ratio of 10:1:1 was added to 85 μL of bee pollen extracts. After 6 min of incubation at room temperature, the absorbance was measured at 593 nm with a PerkinElmer Lambda 365 spectrophotometer (Perkin Elmer Italia, Milano, Italy). The results were expressed as Fe^2+^ equivalents (μmol)/g bee pollen using a water solution of FeSO_4_·7H_2_O (100–2000 μM) for the calibration curve.

### 2.7. Determination of the Cellular Antioxidant Activity in Red Blood Cells (CAA-RBC)

The cellular antioxidant activity of ethanolic bee pollen samples (50 µg/mL, final concentration) was measured by the CAA-RBC assay as previously described by Frassinetti et al. [34]. The fluorescence was measured with a fluorimeter (Perkin-Elmer Victor X3, Perkin Elmer Italia, Milano, Italy) at 485 nm excitation and 535 nm emission and the quercetin (8 µM, final concentration) was used as an antioxidant standard. The antioxidant activity was expressed as CAA unit according to the following formula: CAA unit = 100 − (∫SA/∫CA) × 100 where ∫SA represents the integrated area of the fluorescence curve of the sample, while ∫CA is the control [35]. Quercetin was used as a standard. CAA unit data were derived from five distinct healthy volunteers blood samples and expressed as mean ± standard deviation (SD). One-way ANOVA with Dunnett’s post hoc test: * significantly different from control cells (AAPH-treated cells, CAA = 0), *** *p* < 0.001.

### 2.8. Statistical Analysis

The statistical analysis was performed using GraphPad Prism, version 5.00 for Windows (GraphPad Software, San Diego, CA, USA). Assays were carried out at least in triplicate, and results were expressed as mean values ± standard deviation (SD). Differences in CAA-RBC outcomes were analyzed by one-way analysis of variance (ANOVA) followed by Dunnett’s post-test. A *p*-value lower than 0.05 is considered as statistically significant. Interdependence between the phytochemical profile (1. total phenolics and 2. total flavonoids) and the antioxidant capacity (3. FRAP and 4. CAA-RBC) was evaluated by Pearson’s correlation coefficient (r).

## 3. Results and Discussion

### 3.1. Phytochemical Profile and In Vitro Antioxidant Activity of Bee Pollen Ethanolic Extracts

The phytochemical profile of bee pollen ethanol extracts was determined as total phenolics’ and flavonoids’ content and the results are reported in Table 2. Results shown in Table 2 are also visualized in Figure S1. The total phenolics’ content ranged between 5.78 to 20.15 mg GAE/g of bee pollen extracts. Results were similar to those obtained in references [4,36,37], where values in the ranges 13.53–24.75 mg GAE/g, 7.08–15.27 mg GAE/g, and 12.9–19.8 mg GAE/g were reported, respectively. Higher values were reported by Leja et al. [38] in the range of 12.93–82.43 mg GAE/g.

In the present work, the *Viburnum* (99%) (P03-V) bee pollen extract showed the highest phenolics content, estimated as FC reducing capacity, (20.15 ± 0.15 mg GAE/g fw) followed by *Eucalyptus* (P05-Eu) (19.63 ±2.53 mg GAE/g fw), *Prunus* (P01-P) (18.98 ± 1.36 mg GAE/g fw), and *Brassicaceae* (P01-B) (17.82 ± 1.68 mg GAE/g fw), while *Viburnum* (96%) (P02-V) bee pollen extract showed the lowest level (5.78 ± 0.87 mg GAE/g fw). Total flavonoids content ranged from 7.75 to 23.46 mg CE/g of bee pollen extracts. Results were similar to those obtained by Gabriele et al. [4] and higher than those obtained by Feás et al. [37] and Pascoal et al. [10] (5.91–15.86 mg CE/g, 4.5–7.1 CE/g, 3.71–10.14 CE/g, respectively). The highest level of total flavonoids was found in *Viburnum* (99%) (P03-V) bee pollen extract (23.46 ± 0.08 mg CE/g fw) followed by *Prunus* (P01-P) (22.98 ± 0.24 mg CE/g fw), *Brassicaceae* (P01-B) (21.23 ± 0.08 mg CE/g fw), and *Eucalyptus* (P05-Eu) (21.12 ± 1.53 mg CE/g). At the same time, *Asteraceae* T. (P05-A) showed the lowest levels (7.75 ± 0.62 mg CE/g fw).

Gabriele et al. [4], analyzing a Tuscan polyflora bee pollen, showed similar total phenolics levels and significantly higher flavonoids content than those obtained in the research mentioned above. The differences in the phytochemical composition observed among the different bee pollen samples, especially those belonging to the same plant, might depend not just on their botanical origin but also on other factors, such as climatic conditions and beekeeping activity [39], confirming the great variability on the chemical composition of tested bee pollen samples.

The in vitro antioxidant activity of bee pollen extracts was evaluated by ferric reducing antioxidant power (FRAP), and the results are shown in Table 2. The FRAP assay, in contrast to other tests of antioxidant power, is simple, fast, and robust [40].

The FRAP values of the bee pollen extracts ranged from 14.77 to 190.27 µmol Fe^2+^/g. These results are similar to those obtained by Bilić Rajs et al. [36] and Velásquez et al. [41] with values ranged from 4.51 to 91.19 and 51.97 to 83.56 µmol Fe^2+^/g, respectively. The highest FRAP antioxidant activity was observed in *Prunus* (P01-P) extract with a value of 190.27 ± 8.30 µmol Fe^2+^/g, followed by *Viburnum* (99%) (P03-V) (165.39 ± 6.83 µmol Fe^2+^/g), *Eucalyptus* (P05-Eu) (154.90 ± 8.51µmol Fe^2+^/g), *Brassicaceae* (P01-B) (146.98 ± 1.87 µmol Fe^2+^/g), and *Rubus* (P01-R) (121.85 ± 17.42 µmol Fe^2+^/g), while *Viburnum* (96%) (P02-V) showed the lowest value (14.77 ± 1.27 µmol Fe^2+^/g).

As also visualized in Figure S1, these three experimental methods used to investigate the antioxidant properties of bee pollen extracts (i.e., total phenolics, flavonoids, and FRAP) give rise to coherent results. In all cases, the samples showing the highest antioxidant content and activity are those of *Viburnum* (99%) (P03-V), *Prunus* (P01-P), *Eucalyptus* (P05-Eu), and *Brassicaceae* (P01-B).

### 3.2. Fluorescence Spectroscopic Results

#### 3.2.1. Bulk Analysis

Emission spectra obtained in the bulk of the ten bee pollen samples were first used to identify the main fluorescence spectral regions. Several fluorophores were identified from the characteristic emission/excitation bands and further confirmed by the comparison with the literature and by applying a semi-quantitative deconvolution spectral analysis [30], as described in the following. In particular, the comparison among FFF emission spectra obtained by exciting the samples at λ_ex_ = 280 nm, λ_ex_ = 330–350 nm, and λ_ex_ = 450 nm were used for the first characterization of the spectral profiles. The bee pollen P02-V was used to perform several tests about the reproducibility of the fluorescent spectral profiles, and a selection of emission/excitation spectra of the bee pollen P02-V sample recorded in bulk is reported in the Figure S2.

As a general remark, emission spectra at λ_ex_ = 280 nm are characterized by a band between 320 and 450 nm typical of free amino acids, proteins and phenolic acids, a second band centered between 395 and 450 nm due to vitamins, such as B_6_ and B_9_, and a third large band between 420 and 700 nm, which is more pronounced in samples rich in vitamin B_2_, flavonoids, and pigments. Emission spectra obtained by exciting the sample at λ_ex_ = 350 nm contain a large band between 500 and 600 nm, typical of hydroxycinnamic acids, B vitamins (like B_2_, B_6_, and B_9_), and flavonoids. By fixing λ_ex_ at a wavelength larger than 450 nm, fluorescence spectra are dominated by the emission of flavonoids, such as quercetin, and eventual pigments.

**Figure 1:**
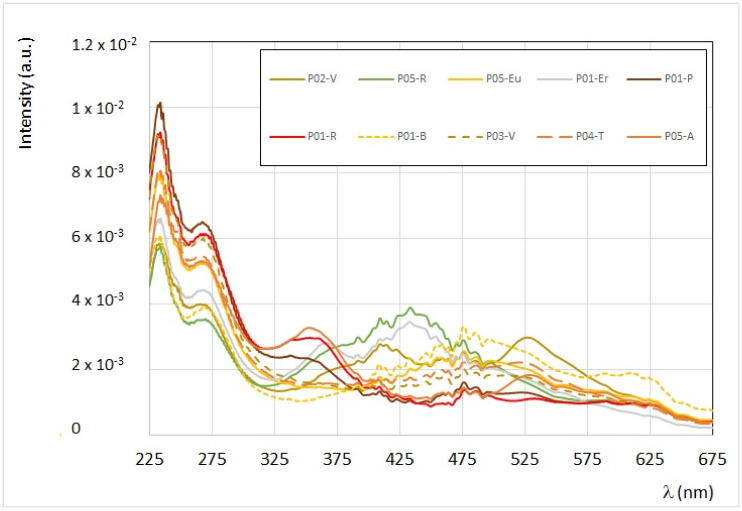
Superposition of synchronous front-face fluorescence (FFF) spectra (Δλ = 60 nm) of the ten bee pollen samples recorded in bulk. The intensity was first corrected by the diffusion contribution [30,32] and then normalized. Sharp signals between 400 and 500 nm are due to the xenon lamp.

This information is contained in the synchronous FFF spectra too, which are used better to identify the presence of eventual superposition among different fluorophores. In Figure 1, the synchronous spectra obtained by fixing the interval Δλ = 60 nm of ten bee pollen samples are reported in the spectral range between 225 to 675 nm.

Three different zones can be identified (see also the Figures S3–S5).

ZONE I (from 225 to 375 nm): this region presents quite sharp peaks which correspond to specific fluorophores. The two bands centered at λ = 230 nm and λ = 270–280 nm (emission at λ = 290 nm and λ = 330–340 nm) are typical of phenolic acids, such as di-hydroxybenzoic acids. The band centered at λ = 290 nm (emission at λ = 350 nm) is mainly due to tryptophan, vanillic, and gallic acids. In contrast, the band at λ = 330–370 nm (emission at λ = 390–430 nm) is typical of niacin (vitamin B_3_), caffeic, and sinapic acids. In this region, the most intense emission is that of *Prunus* (P01-P), *Viburnum 99%* (P03-V), and *Rubus* (P01-R). Some samples present a similar spectral profile: see, for instance, the two synchronous profiles from *Trifolium Pratense (T. pratense)* (P04-T) and *Eucalyptus* (P05-Eu), and the two synchronous profiles from *Rosa sp.* (P05-R) and *Erica* (P01-Er).

ZONE II (from 375 and 500 nm): this region is typical of vitamins (such as B_3_, B_2_, and B_6_), some flavonoids and carotenoids, if present, as further confirmed by UV-Vis spectral absorption of the ethanol extracts (see the following paragraph). In this region, bee pollen samples from *Prunus* (P01-P), *Asteraceae T*. (P05-A), and *Rubus* (P01-R) have the lowest fluorescence intensity. On the other hand, the samples from *Erica* (P01-Er) and *Rosa sp*. (P05-R) present a similar spectral profile with a maximum of intensity at λ = 430–440 nm, corresponding to the emission band at λ = 490–500 nm. The synchronous FFF spectrum of *Brassicaceae* (P01-B) shows the higher emission band at about λ = 530 nm, which corresponds to the typical emission of riboflavin (vitamin B_2_), while the sample from *Viburnum* 96% (P02-V) presents both bands, centered at λ = 310 nm and λ = 470 nm (emission at λ = 370 nm and λ = 530 nm), typical of vitamin B_3_ and B_2_.

ZONE III (from 500 to 700 nm): this region is characterized by a lower intensity, and it is mainly due to flavonoids, and eventual pigments, such as chlorophylls and their derivatives, and anthocyanins. In this region, the most intense emission is observed in *Viburnum* 96% (P02-V), *Asteraceae T.* (P05-A), *Brassicaceae* (P01-B), *Eucalyptus* (P05-Eu), *T. pratense* (P04-T), and *Viburnum* 99% (P03-V), which are those derived from yellow-orange grain pollens, which have a high content in vitamin B_2_. The eventual presence of chlorophylls and their derivatives is low to be detectable even in the green pollen grains of *Rosa sp.* (P05-R). The lowest intensity is that of the white bee pollen of *Erica* (P01-Er).

The assignment and identification of the specific fluorophores in each bee pollen sample were supported by the analysis of the ethanol extracts described in the following paragraph.

#### 3.2.2. Ethanol Extracts Analysis

**Figure 2:**
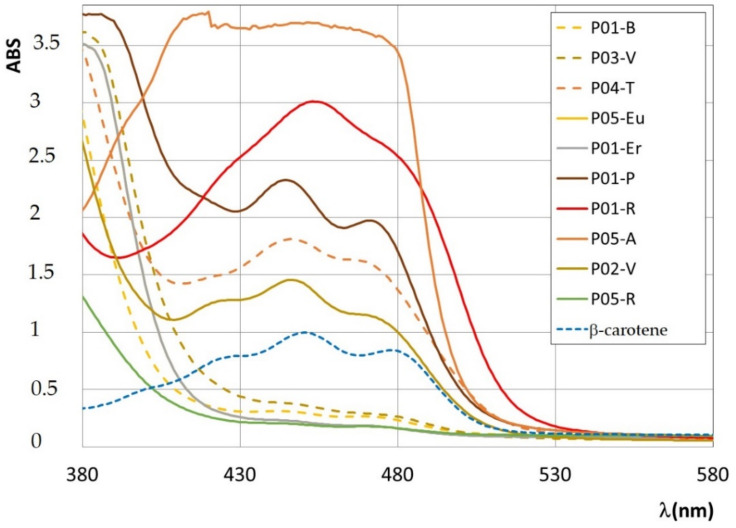
UV-vis absorption spectra of ethanol extracts of the bee pollen samples, as described in the text. The UV-vis spectrum of a solution of β-carotene in ethanol (C = 0.24 mg/mL) is reported for a direct comparison.

The analysis of the spectral features of ethanol extracts derived from the bee pollens allows us to identify fluorophores, which are soluble in ethanol, in particular phenolic acids (hydroxycinnamic and hydroxybenzoic acids), flavonoids, some vitamins, and pigments. Before analyzing the emission/excitation profiles of the extracts, the UV-vis absorption spectra were also recorded in order to identify eventual pigments. The superposition of UV-vis spectra of the ethanol extracts of ten bee pollen samples is reported in Figure 2. From the UV-vis absorption spectra of bee pollen extracts, a quantitative determination of β-carotene’s content was obtained (see also Table S1).

As reported in Table S1, the bee pollen extracts having the higher content of β-carotene are from *Asteraceae T*. (P05-A), *Prunus* (P01-P), *T. pratense* (P04-T), and *Viburnum* (96%) (P02-V). These samples correspond to orange and yellow pollen grains. The sample *Rubus* (P01-R), which has brown pollen grains, has a high content in carotenoids, different from β-carotene, as evident from the peculiar spectral profile (see Figure 2), while the lowest content is that of ethanol extracts from *Rosa sp*. (P05-R) and *Erica* (P01-Er), confirming the previous finding from FFF emission profiles.

**Figure 3:**
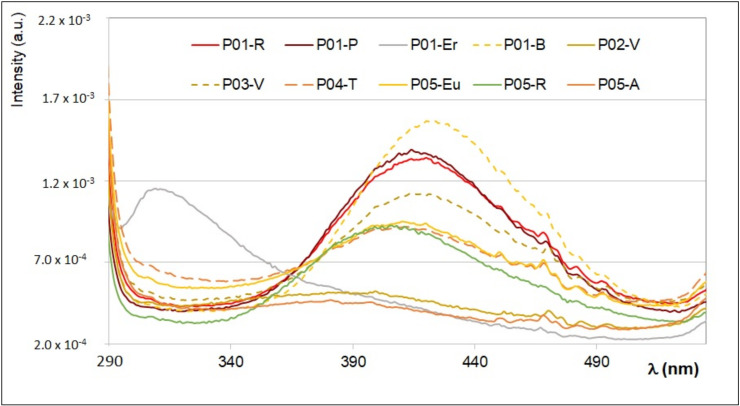
Superposition of the FFF emission spectra of the ethanol extracts of the ten bee pollen samples, prepared as described in the main text, obtained by exciting the sample at λ_ex_ = 280 nm. The intensity was normalized.

In Figure 3, the superposition among FFF emission spectra of the ethanol extracts of the ten bee pollen samples obtained by exciting the sample at λ_ex_ = 280 nm is reported.

**Figure 4:**
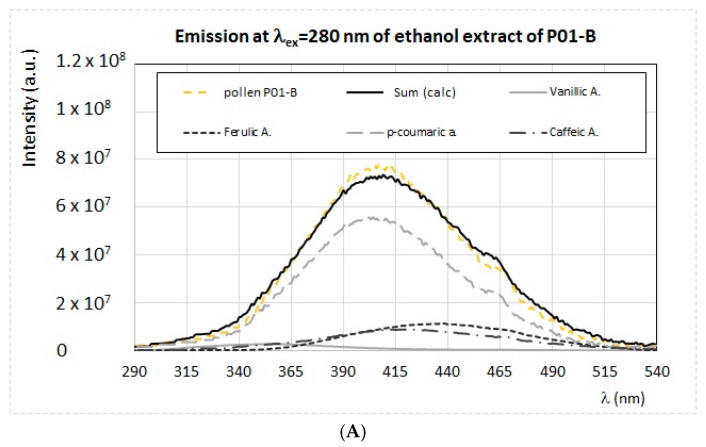

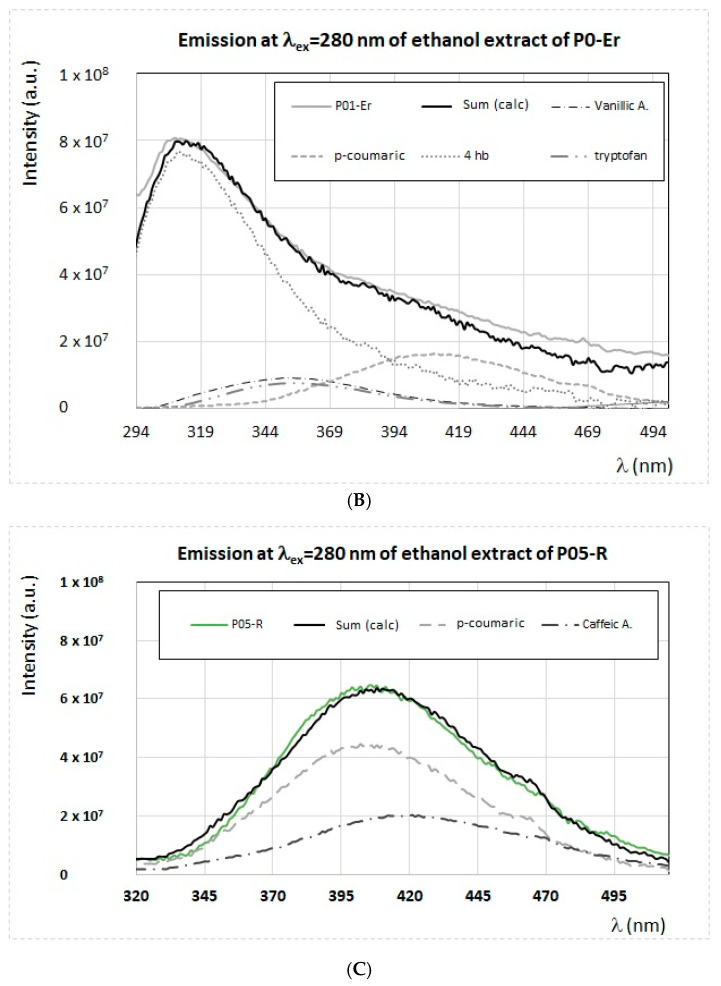
(**A**–**C**) Experimental and calculated FFF emission spectra of the ethanol extracts obtained from the samples P01-B (**A**), P01-Er (**B**), and P05-R (**C**), prepared as described in the main text, obtained by exciting the sample at λ_ex_ = 280 nm. The calculated spectra, indicated as Sum (calc), are obtained as the sum of different components: ferulic acid, p-coumaric acid, caffeic acid, vanillic acid, 4-hydroxybenzoic acid (4 hb), and tryptophan.

By exciting the ethanol extracts at λ_ex_ = 280 nm, it is possible to recognize typical emission bands ranging between 320 and 500 nm, due to phenolic acids, such as gallic, caffeic, ferulic, p-coumaric syringic, and vanillic acids, as well as the protein and aminoacidic content (namely tryptophan). The presence of these fluorophores was confirmed by the semi-quantitative simulation of the emission spectra, already described in previous works [28,29,30], obtained by considering the additive contribution of each standard fluorophores. As an example, in Figure 4A–C, the superposition between the experimental and simulated emission spectrum of three ethanol extracts obtained at λ_ex_ = 280 nm, quite representative of all samples, is reported together with the contribution of the single fluorophores.

The emission spectrum of the ethanol extract from *Brassicaceae* (P01-B) has the highest intensity, and it is due to the contribution from vanillic, ferulic, caffeic, and p-coumaric acids (see Figure 4A). The extract from *Erica* (P01-Er) has a very different emission profile (see Figure 4B), and it is well reproduced by considering as main fluorophore the 4-hydroxybenzoic acid, with the addition of vanillic and p-coumaric acids and tryptophan in much less amount. All other samples show an emission profile with spectral features between those of *Brassicaceae* (P01-B) and those of *Rosa sp*. (P05-R), which is dominated by the presence of p-coumaric and caffeic acids.

**Figure 5:**
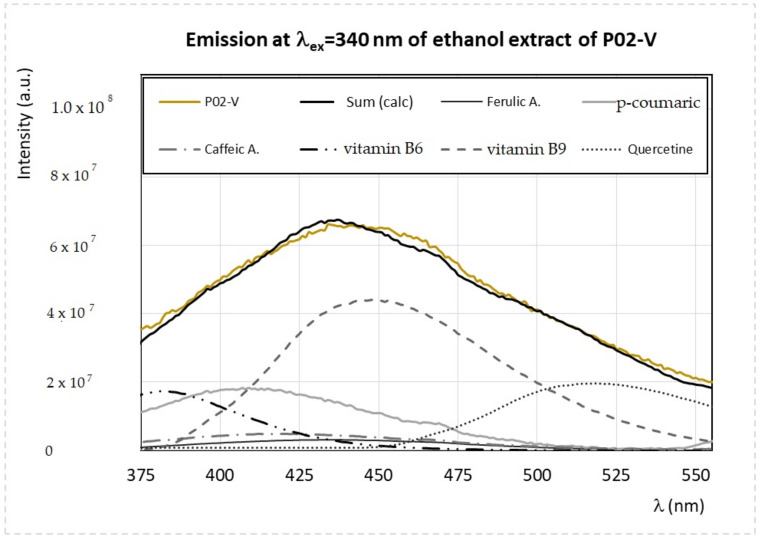
Experimental and calculated FFF emission spectra of the ethanol extracts obtained from the samples P02-V, prepared as described in the main text, obtained by exciting the sample at λ_ex_ = 340 nm. The calculated spectrum, indicated as Sum (calc), is obtained as the sum of different components: ferulic acid, p-coumaric acid, caffeic acid, vitamin B_6_, vitamin B_9_, and quercetin.

**Figure 6:**
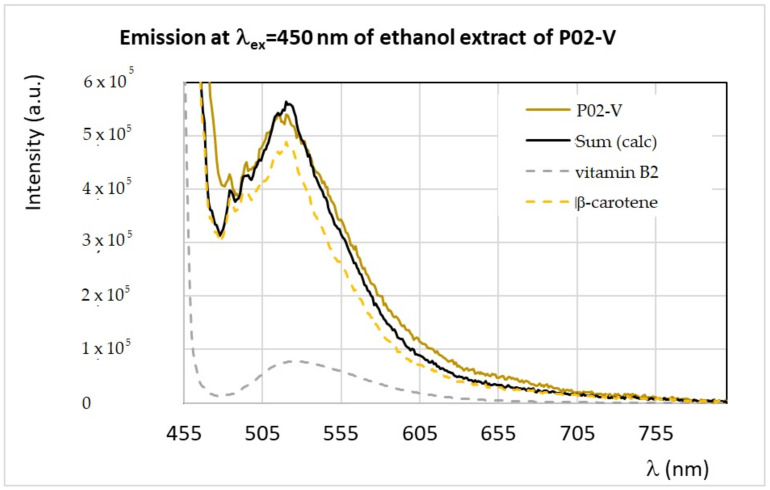
Experimental and calculated FFF emission spectra of the ethanol extracts obtained from the samples P02-V, prepared as described in the main text, obtained by exciting the sample at λ_ex_ = 450 nm. The calculated spectrum, indicated as Sum (calc), is obtained as the sum of different components: vitamin B_2_ (riboflavin) and β-carotene.

Analogously, it is possible to analyze the emission spectra of ethanol extracts obtained at λ_ex_ = 340 nm and λ_ex_ = 450 nm, which confirmed the presence of vitamins (B_2_, B_6_, and B_9_), flavonoids such as quercetin, and carotenoids. As an example, the simulated versus experimental spectra of the *Viburnum* 96% pollen sample (P02-V) recorded at λ_ex_ = 340 nm, and λ_ex_ = 450 nm are reported in Figure 5 and Figure 6, respectively.

Among the analyzed bee pollen ethanol extracts, those showing the highest emission intensity when excited at λ_ex_ = 340 nm and λ_ex_ = 450 nm are those from *Brassicaceae* (P01-B), *Rosa sp.* (P05-R), and *Prunus* (P01-P), which correspond, according to the spectral simulation, to a high content in vitamin B_9_ and vitamin B_6_, as well as in flavonoids such as quercetin.

It is interesting to note that the bee pollen extracts showing the highest intensity in fluorescence emission are those obtained from *Prunus* (P01-P) and *Brassicaceae* (P01-B), which are the samples showing the highest antioxidant properties, as reported in the previous Section 3.1.

### 3.3. Bee Pollen Biological Activity on Ex Vivo Human Erythrocytes

The biological activity of 50 µg/mL bee pollen extracts was analyzed by cellular antioxidant activity in red blood cells (CAA-RBC) [34] under oxidative condition, and results are shown in Table 3 and visualized in Figure S6. All treatments, as well as the 8 µM quercetin (83.37 ± 2.23 CAA unit) used as a standard, had significantly enhanced the cellular antioxidant activity of human erythrocytes compared to control cells that were only treated with AAPH (CAA unit = 0). Particularly, the CAA-RBC assay revealed that *Eucalyptus* (P05-Eu) (54.61 ± 8.51 CAA unit) displayed the highest cellular antioxidant activity, followed by *Rubus* (P01-R) (52.69 ± 12.57 CAA unit), *Prunus* (P01-P) (40.71 ± 8.92 CAA unit), *Viburnum* (99%) (P03-V) (39.47 ± 8.09 CAA unit), and *Asteraceae* T. (P05-A) (38.24 ± 6.77 CAA unit), while *Erica* (P01-Er) (27.22 ± 6.99 CAA unit) showed the lowest erythrocytes antioxidant protection.

One-way analysis of variance with Dunnett’s post hoc test showed significantly higher CAA values following all bee pollen extract and quercetin pre-treatments with respect to the control cells (*p* < 0.001), underlying a significant contribution of bee pollen extracts in the erythrocytes antioxidant protection.

Pearson’s correlation analysis was performed to explore the relationship between total phenolics content, flavonoids, FRAP, and CAA-RBC results of all analyzed bee pollen samples. As shown in Table 4, Pearson’s analysis revealed a positive interdependence between total phenolics content, flavonoids, FRAP, and CAA-RBC data. As expected, a strong correlation was found between total phenolics and flavonoids (r = 0.8891, *p* < 0.001), as well as between total phenolics and FRAP in vitro activity (r = 0.9602, *p* < 0.001), which also correlated with the flavonoids content (r = 0.9512, *p* < 0.001). Finally, a moderate but significant correlation was observed between total phenolics and CAA-RBC ex vivo activity (r = 0.6484, *p* < 0.05).

These results showed for the first time the antioxidant activity of bee pollen in an ex vivo system involving red blood cells. Further studies could be useful to better investigate the intracellular pathways involved in the bee pollen antioxidant response.

## 4. Conclusions

As a final remark, in the present study, the botanical origin, phytochemical profile, in vitro and ex vivo antioxidant activity of ten bee pollen samples from Tuscany (Italy) were investigated. Each sample was analyzed by means of FFF spectroscopy both in the bulk and the ethanol extract form in order to identify the main fluorophores responsible for the emission properties. Moreover, this study allowed us to put in evidence differences and analogies among pollens with a different botanical origin in terms of typical FFF profiles. The semi-quantitative analysis of FFF emission spectra revealed the presence of specific hydrosoluble vitamins, such as B_2_, B_3_, B_6_, and B_9_, amino acids, such as tryptophan, phenolics acids, such as hydroxycinnamic and hydroxybenzoic acids, flavonoids, such as quercetin, and natural pigments, such as carotenoids and chlorophylls’ derivatives. The results obtained in this work indicate that Tuscan bee pollen samples are rich in phytochemical compounds displaying good antioxidant in vitro activity. In fact, the experimental investigations of the antioxidant properties of bee pollen extracts, namely the total phenolics, flavonoids, and FRAP test, are coherent and show that the bee pollen samples with the highest in vitro antioxidant activity are those showing the most intense fluorescence emission and higher content in bioactive chemical compounds. Moreover, all bee pollen samples are capable of protecting ex vivo human erythrocytes from AAPH-induced oxidation, as shown from the CAA-RBC test reported for the first time in this work on bee pollen extracts. All these results make Tuscan bee pollen as a potential nutraceutical product and an excellent dietary supplement useful for free radical associated disease prevention.

## Figures and Tables

**Table 1:** List of the bee pollen samples investigated in this work. The sample label reflects the sample of origin (P0X) followed by one of two letters to indicate their botanical origin. The botanical origin is expressed in %, the geographical origin, the color, and year and month of harvesting are also reported. Each pollen load owned a homogeneous and monospecific pollen content.

Sample Label	Botanical Origin (%)	Geographical Origin	Colour	Year and Month of Harvest
P01-P	*Prunus* (70%)	Garfagnana (LU)	Brown	April 2017
P01-Er	*Erica* (96%)	Garfagnana (LU)	White	April 2017
P01-B	*Brassicaceae* (94%)	Garfagnana (LU)	Yellow	April 2017
P01-R	*Rubus* (90%)	Garfagnana (LU)	Red	April 2017
P02-V	*Viburnum* (96%)	Garfagnana (LU)	Yellow	October 2016
P03-V	*Viburnum* (99%)	Garfagnana (LU)	Yellow	March 2016
P04-T	*Trifolium pratense (T. pratense)* (84%)	Garfagnana (LU)	Yellow	April 2016
P05-A	*Asteraceae T.* (100%)	Caniparola-Fosdinovo (MS)	Orange	April 2016
P05-Eu	*Eucalyptus* (96%)	Caniparola-Fosdinovo (MS)	Yellow	April 2016
P05-R	*Rosa Sp.* (100%)	Caniparola-Fosdinovo (MS)	Green	April 2016

**Table 2:** Total phenolics, flavonoids, and ferric reducing antioxidant power (FRAP) assay results of bee pollen ethanol extracts. Data are expressed as mean ± standard deviation (SD) of three determinations.

Sample Label	Total Phenolics (mg GAE/g fw)	Flavonoids (mg CE/g fw)	FRAP (µmol Fe^2+^/g)
(P01-P)	18.98 ± 1.36	22.98 ± 0.24	190.27 ± 8.30
(P01-Er)	8.23 ± 0.51	12.19 ± 0.12	40.90 ± 0.63
(P01-B)	17.82 ± 1.68	21.23 ± 0.08	146.98 ± 1.87
(P01-R)	14.15 ± 1.03	16.72 ± 0.45	121.85 ± 17.42
(P03-V)	20.15 ± 0.15	23.46 ± 0.08	165.39 ± 6.83
(P02-V)	5.78 ± 0.87	10.07 ± 0.14	14.77 ± 1.27
(P04-T)	10.71 ± 0.46	16.34 ± 0.28	73.32 ± 1.71
(P05-A)	11.41 ± 1.03	7.75 ± 0.62	40.04 ± 2.54
(P05-Eu)	19.63 ± 2.53	21.12 ± 1.53	154.90 ± 8.51
(P05-R)	11.49 ± 0.65	12.36 ± 0.33	70.52 ± 1.28

**Table 3:** Cellular antioxidant activity in red blood cells (CAA-RBC) results of ethanol bee pollen extracts. CAA unit data were derived from five distinct healthy volunteers’ blood samples and expressed as mean ± standard deviation (SD).

Sample Label	CAA-RBC (CAA Unit)
(P01-P)	40.71 ± 8.92
(P01-Er)	27.22 ± 6.99
(P01-B)	37.03 ± 9.30
(P01-R)	52.69 ± 12.57
(P03-V)	39.47 ± 8.09
(P02-V)	30.97 ± 6.28
(P04-T)	30.86 ± 7.19
(P05-A)	38.24 ± 6.77
(P05-Eu)	54.61 ± 8.51
(P05-R)	34.78 ± 8.28

**Table 4:** Linear correlation coefficients (r) between total phenolics, total flavonoids, ferric reducing antioxidant power (FRAP), and cellular antioxidant activity (CAA-RBC) data of analyzed bee pollens (*n* = 10) considered as a unique sample. Pearson correlation, two-tailed: * *p* < 0.05; **** *p* < 0.0001. ^ns^ = not statistically significant.

	Pearson Coefficients (r)			
	*1.*	*2.*	*3.*	*4.*
1. Total phenolics	1			
2. Total flavonoids	0.8891 ****	1		
3. FRAP in vitro activity	0.9602 ****	0.9512 ****	1	
4. CAA-RBC ex vivo activity	0.6484 *	0.4549 ^ns^	0.6088 ^ns^	1

## Supplementary Materials

The following are available online at https://www.mdpi.com/2076-3921/9/10/1001/s1, Figure S1: total phenolics, flavonoids concentration and FRAP assay results of bee pollen ethanol extracts; Figure S2: a selection of FFF spectra of P02-V (test of reproducibility, emission/excitation spectra); Figures S3–S5: enlargement of Figure 1 (three zones of the synchronous spectrum); Figure S6: CAA-RBC assay results of ethanol bee pollen extracts; Table S1: concentration of β-carotene of ethanol extracts of bee pollen samples from the analysis of the UV-vis spectra.
