# Supplementary material for: Antioxidant, Nutraceutical Properties, and Fluorescence Spectral Profiles of Bee Pollen Samples from Different Botanical Origins
Source: Antioxidants (Basel). 2020 Oct 15;9(10):1001. doi: 10.3390/antiox9101001 (PMC7602780; doi:10.3390/antiox9101001)
Supplement: Supplementary file 1 [file antioxidants-09-01001-s001.pdf]

# Supplementary Materials

## Antioxidant, Nutraceutical Properties, and Fluorescence Spectral Profiles of Bee Pollen Samples from Different Botanical Origins

**Daniele Barbieri [1,2,†], Morena Gabriele [1,†], Martina Summa [2], Raffaele Colosimo [3], Donatella Leonardi [4], Valentina Domenici [2,*] and Laura Pucci [1]**

[1] National Research Council (CNR), Institute of Agricultural Biology and Biotechnology, Via Moruzzi 1, 56124 Pisa, Italy; danielebarbieri.bd@gmail.com (D.B.); gabriele.morena@hotmail.it (M.G.); lauric16@yahoo.it (L.P.)
[2] Chemistry and Industrial Chemistry Department, University of Pisa, Via Moruzzi 13, 56124 Pisa, Italy; m.summa1@studenti.unipi.it
[3] Quadram Institute Bioscience, Norwich Research Park, Norwich, Norfolk, NR4 7UQ, UK; raffaele.colosimo@quadram.ac.uk
[4] Department of Biology, University of Rome "Tor Vergata", Via della Ricerca Scientifica 1, 00133 Roma, Italy; leonardi@uniroma2.it

* Correspondence: valentina.domenici@unipi.it; Tel.: +39-050-2219-215
† The authors contributed equally to this study.

**Phytochemical profile and in vitro antioxidant activity of bee pollen ethanolic extracts:**

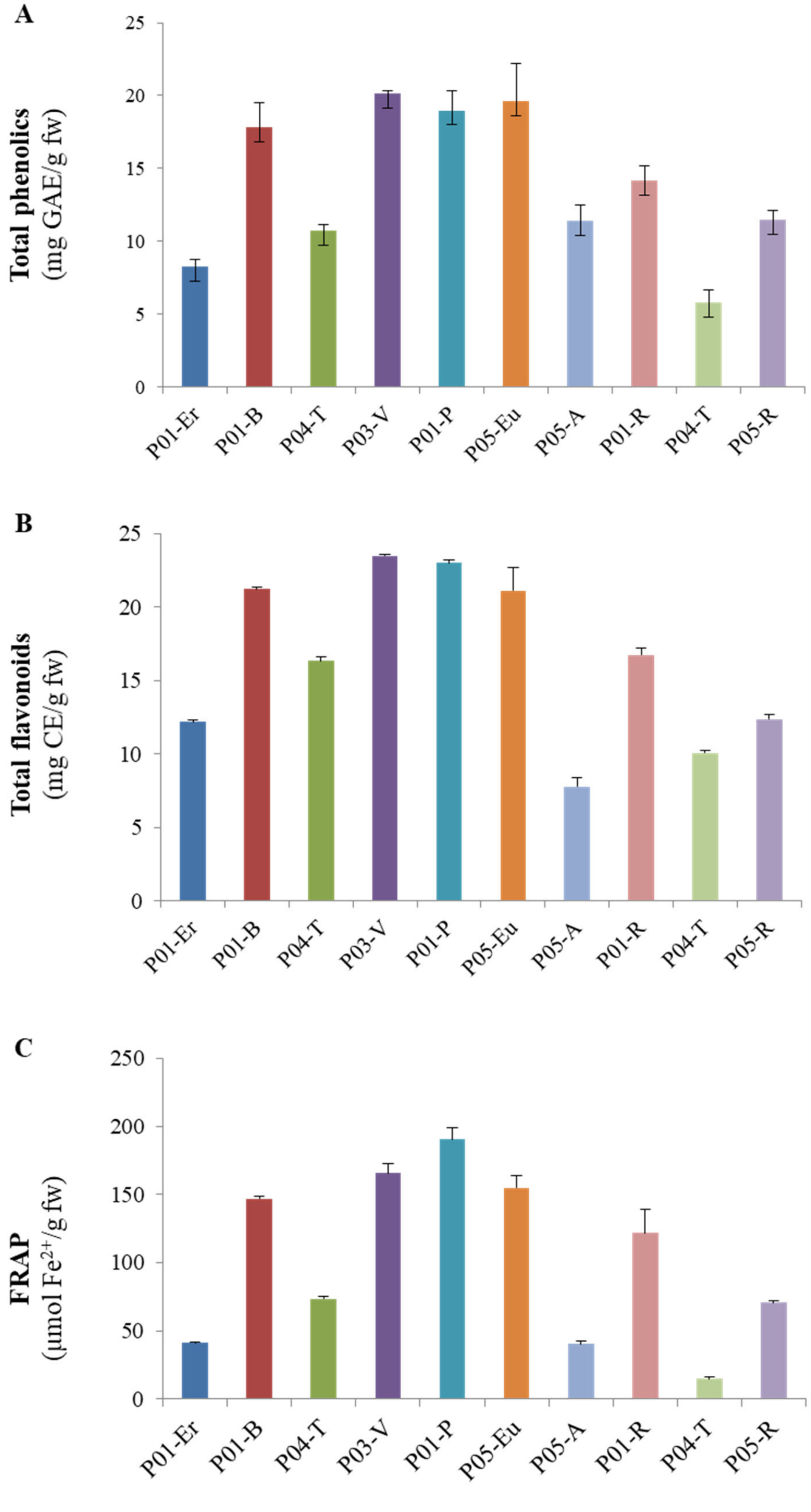


**Figure S1:** Total phenolics, flavonoids concentration and FRAP assay results of bee pollen ethanol extracts.

**Focus on the pollen P02-V in the bulk (test of reproducibility and effect of the conservation time):**

The pollen P02-V was used to test the reproducibility of the FFF spectroscopic approach. Four different samples were prepared (from a to d) using powders prepared in different period (time interval of 6 about months). Three samples (P02-V (a), P02-V (b) and P02-V (c)) were prepared from the same powder, which was obtained as described in the main text, from about 20 pollen grains of P02-V. P02-V (d) is obtained from a powder prepared from about 10 pollen grains, prepared about six months before the previous powder.

The test is important in order to verify that the spectral profile of the sample is not affected by different pollen grains of origin and that the main features of the spectra are not changed during time. In particular, the position of the emission and excitation bands is the same in all samples.

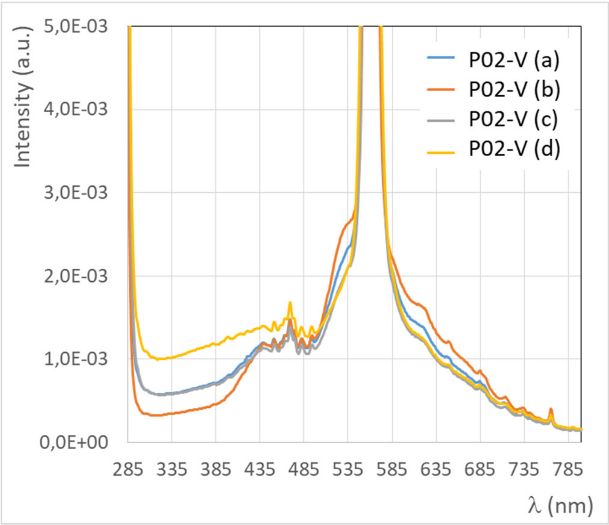


**(A)**

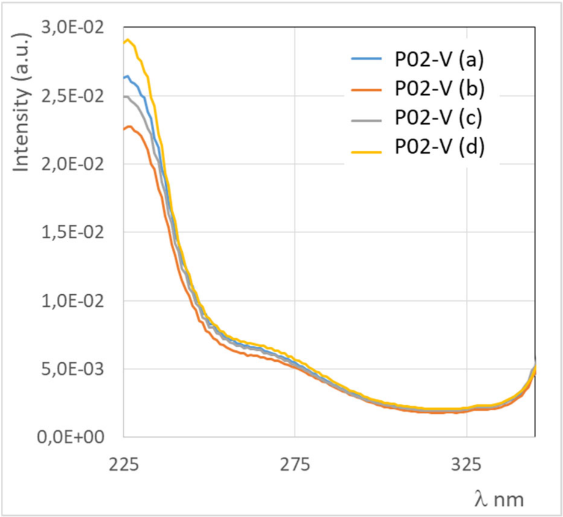


**(B)**

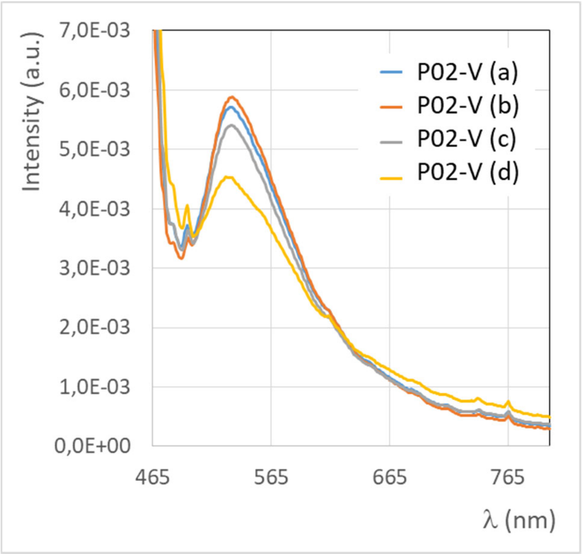


**(C)**

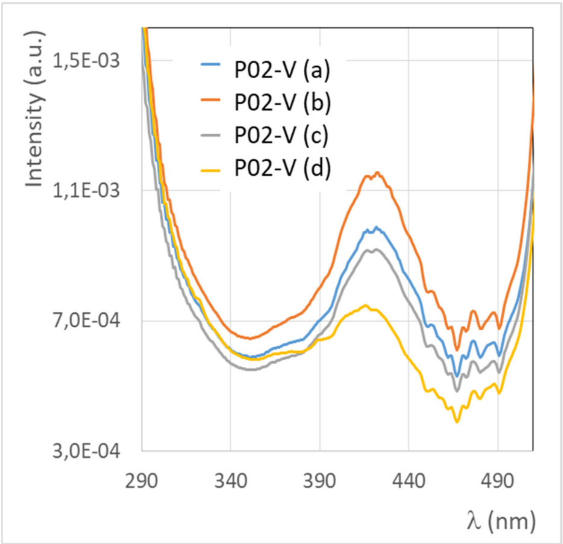


**(D)**

**Figure S2:** Superposition of FFF emission and excitation spectra of four samples obtained from the bee pollen P02-V, recorded in the bulk without any treatment. (A) Emission spectra with $\lambda_{ex}$=280 nm. (B) Excitation spectra with $\lambda_{em}$=360 nm. (C) Emission spectra with $\lambda_{ex}$=450 nm. (D) Excitation spectra with $\lambda_{ex}$=530 nm. Samples P02-V (a), P02-V (b) and P02-V (c) are obtained from the same powder prepared from about 20 pollen grains; P02-V (d) is obtained from a powder prepared from about 10 pollen grains, prepared about six months before the previous powder. Intensity was normalized. Sharp signals between 400 and 500 nm in the emission spectra are due to the xenon lamp.

**Selection of different parts of the synchronous spectra of ten bee pollen samples:**

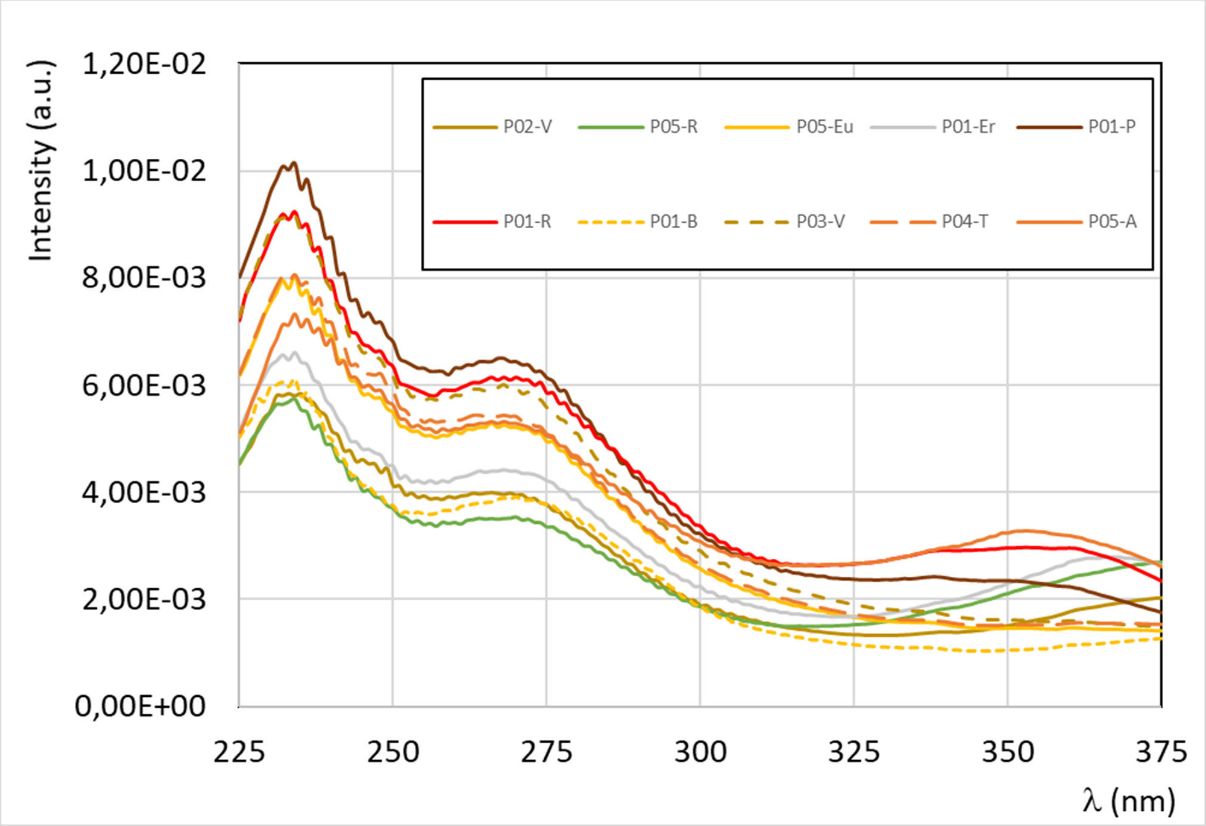


**Figure S3:** Selection of the superposition of FFF synchronous spectra (Δλ=60 nm) of the ten bee pollen samples recorded in the bulk in the spectral range from 225 to 375 nm. Intensity was first corrected by the diffusion contribution and then normalized.

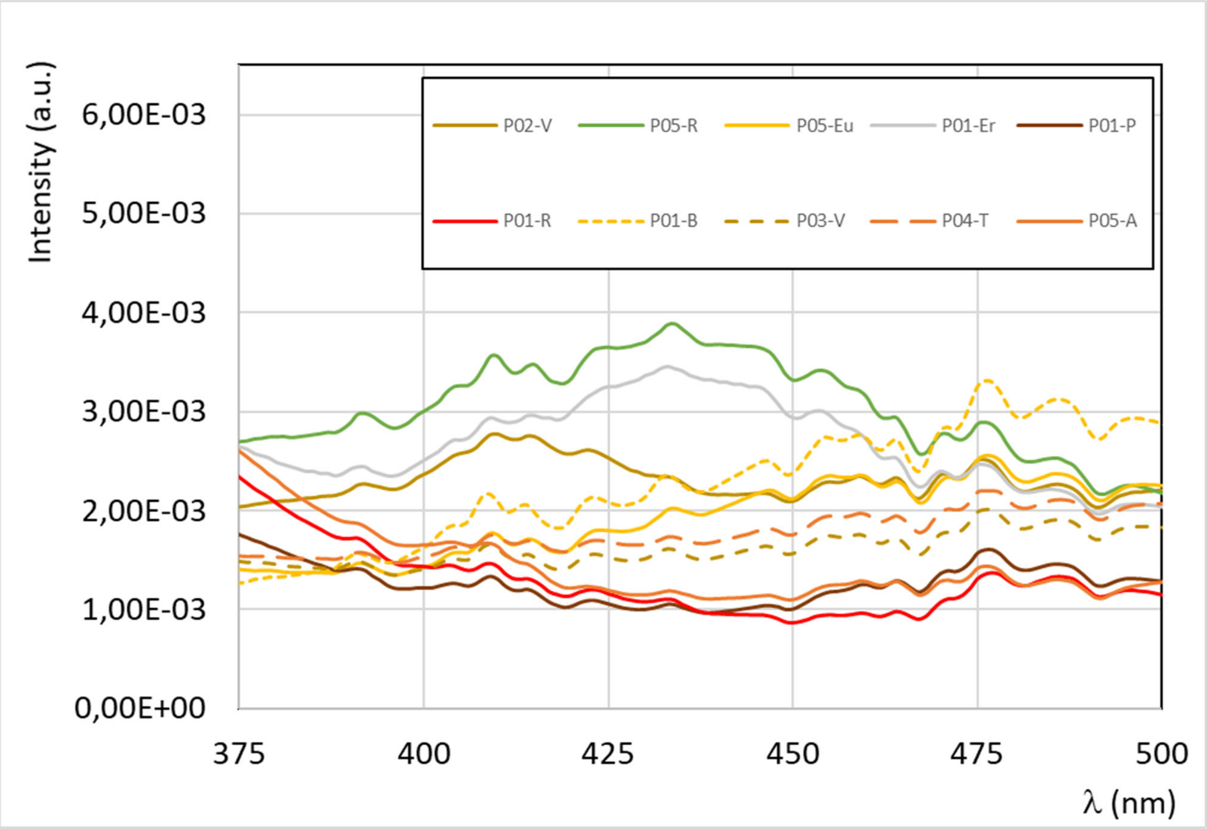


**Figure S4:** Selection of the superposition of FFF synchronous spectra (Δλ=60 nm) of the ten bee pollen samples recorded in the bulk in the spectral range from 375 to 500 nm. Intensity was first corrected by the diffusion contribution and then normalized. Sharp signals between 400 and 500 are due to the xenon lamp.

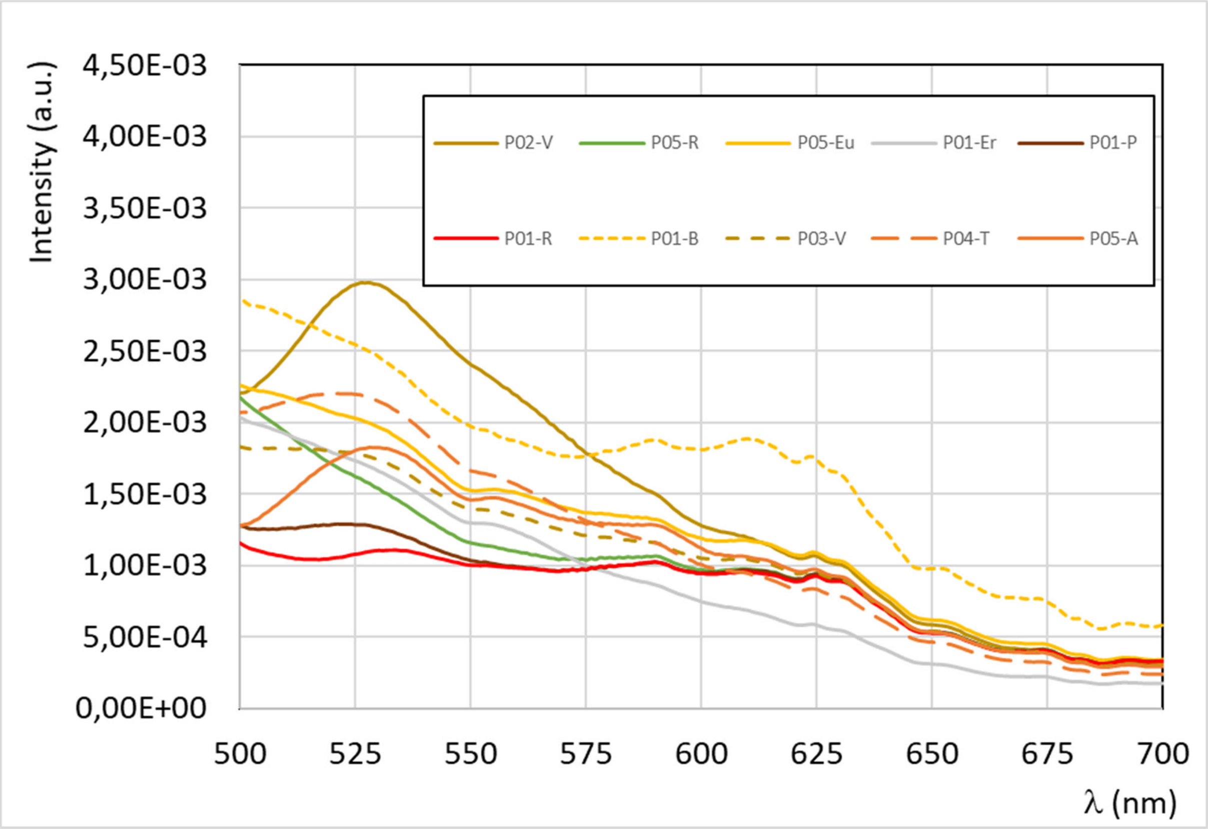


**Figure S5:** Selection of the superposition of FFF synchronous spectra (Δλ=60 nm) of the ten bee pollen samples recorded in the bulk in the spectral range from 375 to 700 nm. Intensity was first corrected by the diffusion contribution and then normalized.

## Extracts of bee pollen samples in ethanol solvent:

***UV-vis Absorbance spectra of ethanol extracts***

**Table S1:** Concentration of β-carotene in the ethanol extracts of the ten bee pollen samples, as evaluated from the comparison with the absorbance spectrum of a solution of β-carotene in ethanol.

| **Sample label** | **Concentration of β-carotene in the ethanol extracts** |
|---|---|
| *P01-P* | 0.52±0.02 mg/ml |
| *P01-Er* | 0.05±0.02 mg/ml[a] |
| *P01-B* | 0.10±0.02 mg/ml |
| *P01-R* | 0.66±0.02 mg/ml[c] |
| *P02-V* | 0.32±0.02 mg/ml |
| *P03-V* | 0.13±0.02 mg/ml |
| *P04-T* | 0.43±0.02 mg/ml |
| *P05-A* | 1.14±0.02 mg/ml[b] |
| *P05-Eu* | 0.07±0.02 mg/ml |
| *P05-R* | 0.04±0.02 mg/ml[a] |

[a] values close to the limit of quantification

[b] This value was determined after diluting the original extracts.

[c] The spectral profile reveals the presence of additional carotenoids with respect to β-carotene.

**Phytochemical profile and in vitro antioxidant activity of bee pollen ethanolic extracts:**

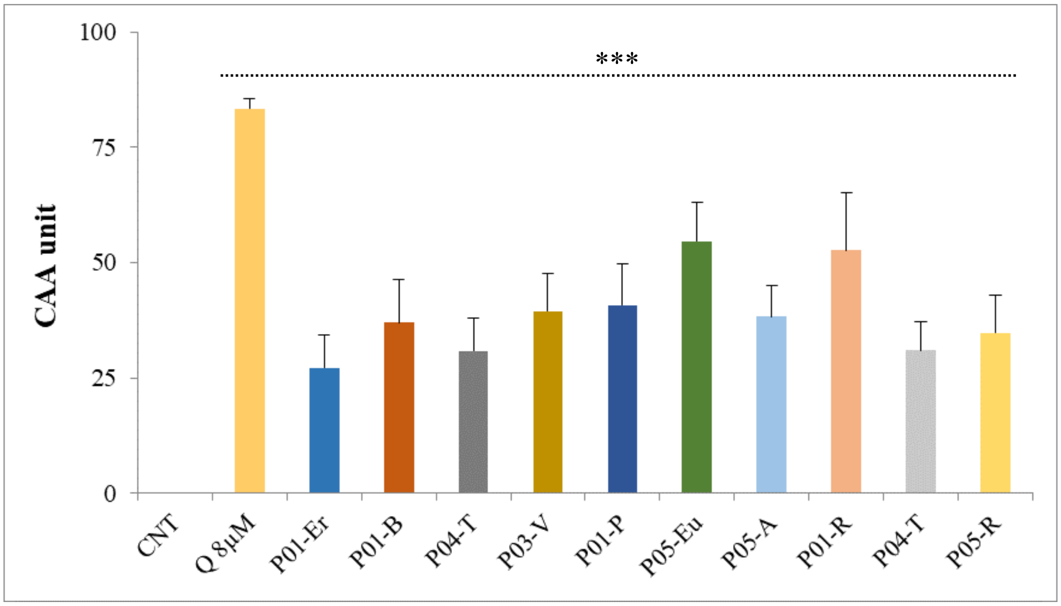


**Figure S6:** CAA-RBC assay results of ethanol bee pollen extracts.
